# Association between oral microbiota and inflammatory bowel disease: A 2-sample Mendelian randomization study in East Asian populations

**DOI:** 10.1097/MD.0000000000047909

**Published:** 2026-03-06

**Authors:** Rong-Xin Xie, Xin-Yu Ci, Yu-Bao Xue, Mei Liu, Mei-Juan Zhang

**Affiliations:** a Department of Gastroenterology, The First Affiliated Hospital of Shandong First Medical University & Shandong Provincial Qianfoshan Hospital, Shandong First Medical University, Jinan, Shandong, China; b Department of GasTroenterology, The First Affiliated Hospital of Shandong First Medical University & Shandong Provincial Qianfoshan Hospital, Jinan, Shandong, China; c Department of General Practice, The First Affiliated Hospital of Shandong First Medical University & Shandong Provincial Qianfoshan Hospital, Shandong Engineering Laboratory for Health Management, Jinan, Shandong, China.

**Keywords:** causality, inflammatory bowel disease, Mendelian randomization, oral microbiome

## Abstract

Inflammatory bowel disease (IBD), including ulcerative colitis (UC) and Crohn’s disease (CD), is increasingly prevalent in East Asia. Its pathogenesis is linked to microbiota dysbiosis via the oral-gut axis, but population-specific causal evidence remains scarce. This study aimed to clarify the causal associations between oral microbiota and UC/CD in East Asian populations using Mendelian randomization (MR), providing evidence for IBD etiology and precise prevention/treatment. A 2-sample MR approach was adopted, using genome-wide association study data of East Asian populations. Single nucleotide polymorphisms associated with tongue dorsum and salivary microbiota were selected as instrumental variables after rigorous screening (*F*-statistic > 10, linkage disequilibrium R^2^ = 0.001). Inverse variance weighted was the primary analysis method, supplemented by sensitivity tests (MR-PRESSO, MR-Egger intercept test, etc) and Benjamini–Hochberg multiple testing correction (false discovery rate < 0.05). A total of 82 oral microbiota taxa (22 families, 35 genera) were significantly causally associated with UC (FDR < 0.05), and 21 taxa (10 families, 12 genera) with CD (FDR < 0.05). High-risk taxa included Aggregatibacter and Streptococcus (OR > 1), while protective taxa included Fusobacterium_periodonticum_C_mgs_3022 and TM7x_unclassified_mgs_1084 (OR < 1). A distinct “mixed effect” was identified: the Streptococcus genus was risky for UC but protective for CD; genera such as Streptococcus (UC), Oribacterium (CD), and TM7x (CD) exhibited bidirectional risk/protective associations within a single IBD subtype; and the TM7x genus was risky for UC and showed bidirectional effects in CD. Other genera (e.g., Fusobacterium, Aggregatibacter) only had unidirectional associations. This study is the first to confirm the causal association between oral microbiota and IBD in East Asian populations, revealing the heterogeneity and “mixed effect” of this association. Identified high-risk and protective oral microbiota taxa provide new insights into IBD etiology and potential targets for clinical precise prevention and treatment.

## 1. Introduction

Inflammatory bowel disease (IBD) is a group of complex diseases characterized by chronic nonspecific intestinal inflammation, mainly including ulcerative colitis (UC) and Crohn’s disease (CD).^[1]^ In recent years, the incidence of IBD has continued to rise in East Asia, and the disease burden has increasingly intensified.^[2]^ Its pathogenesis is closely related to host genetics, environment, and microbiota dysbiosis.^[3]^ Intestinal microbiota imbalance is considered a key link in the pathogenesis of IBD,^[4]^ but traditional observational studies have difficulty clarifying the causal relationship between microbiota and the disease.^[5]^

The proposal of the “oral-gut axis” concept has revealed the potential association between oral microbiota and intestinal diseases.^[6]^ Oral microbiota can ectopically colonize the intestine through the digestive tract and induce intestinal inflammation.^[7,8]^ However, most existing studies are animal experiments or observational analyses, lacking population-level causal evidence. Moreover, the population specificity of microbiota composition makes it difficult to extrapolate results from Western populationsto East Asians.^[9]^ Mendelian randomization (MR) uses genetic variations as instrumental variables, which can effectively avoid confounding bias and reverse causation, providing strong support for causal inference between microbiota and diseases.^[10,11]^ Currently, MR studies on oral microbiota and IBD in East Asian populations are still lacking.

This study adopted a 2-sample MR method to systematically explore the causal associations between oral microbiota (tongue dorsum and salivary microbiota) and UC/CD based on East Asian GWAS data. Through strict selection of instrumental variables, combined with multiple sensitivity analyses and multiple testing corrections, the association characteristics and heterogeneity between oral microbiota and IBD were clarified, providing scientific evidence for the etiological research and precise prevention and treatment of IBD in East Asian populations.

## 2. Materials and methods

### 2.1. Source of exposure data

This study conducted a 2-sample MR analysis, using single nucleotides polymorphisms (SNPs) associated with oral microbiota as instrumental variables (IVs). The analysis utilized summary statistics from a pioneering genome-wide association study (GWAS) that explored oral microbiota characteristics in an East Asian cohort. Detailed data sources and sequencing methods for tongue dorsum and salivary microbiota have been described in the study by Dong et al.^[12]^

### 2.2. Source of outcome data

The GWAS data for ulcerative colitis and Crohn’s disease used in this analysis were obtained from the IEU OpenGWAS project. The UC cohort included 17,689 participants (314 patients and 17,375 controls), and the CD cohort included 5409 participants (1690 patients and 3719 controls). The exposure and outcome datasets do not share participants.

### 2.3. Selection of instrumental variables

In this MR study, SNPs closely associated with each oral microbiota taxon were selected as instrumental variables. Due to the limited number of IVs screened using a strict *P*-value threshold (*P* < 5E−08), a more lenient criterion (*P* < 1E−05/5E−06) was adopted to collect more IVs, thereby enhancing the reliability of the study results. To ensure the independence of each IV and reduce the impact of linkage disequilibrium, data from the 1000 Genomes Project East Asian population^[13]^ were used to prune SNPs within a 10,000 kb range, with a linkage disequilibrium coefficient R^2^ threshold of 0.001. To eliminate the impact of weak instrumental variable bias on the estimation of association effects, SNPs with an *F*-statistic greater than 10 were selected. The formula for calculating the *F*-statistic is: *F* = (*R*^2^/(1 − *R*^2^)) × ((n − *k* − 1)/*k*), where *R*^2^ represents the proportion of variance explained by the IVs for each oral microbiota component, n is the sample size, and *k* is the number of IVs. *R*^2^ was calculated using the minor allele frequency (MAF) and effect estimate (β) with the formula: *R*^2^ = 2 × MAF × (1 − MAF) × (β/SD)^2^. An *F*-statistic >10 indicates that the results are not affected by weak instrumental variables.^[14]^

### 2.4. Mendelian randomization analysis

**Figure 1.**
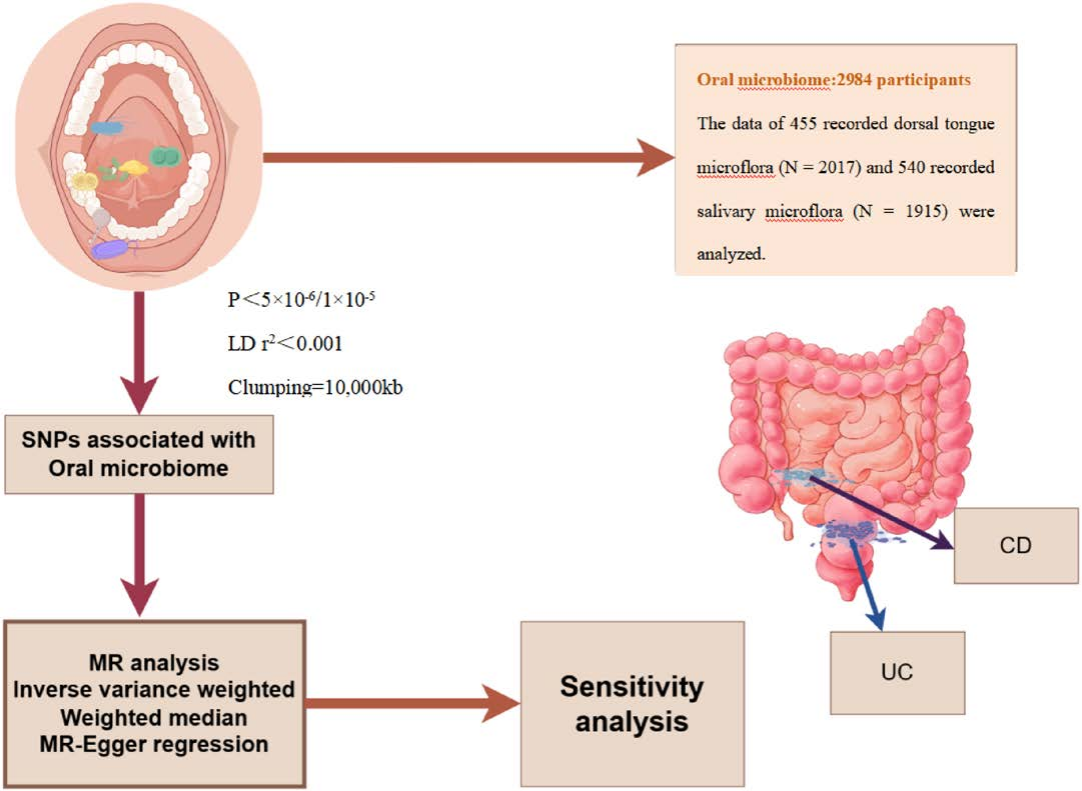
Flowchart for the study of the association between oral microbiota and IBD. CD = Crohn's disease, IBD = inflammatory bowel disease, LD = linkage disequilibrium, MR = Mendelian randomization, SNP = single nucleotide polymorphism, UC = ulcerative colitis.

Data analysis was performed using R 4.3.1 software and the TwoSampleMR (0.5.6) package (University of Auckland, Auckland, Auckland Region, New Zealand). MR analysis aimed to explore the potential causal association between oral microbiota exposure and IBD. The analysis mainly adopted the inverse variance weighted method,^[14]^ with the weighted median method and MR-Egger regression as auxiliary analysis tools.^[15,16]^ When the number of SNPs was small due to insufficient sample size of the outcome variable, the Wald ratio method was used for analysis. Statistical significance was set at *P* < .05 (see Fig. 1).

### 2.5. Sensitivity analysis

To verify the accuracy and robustness of the above MR analysis results, sensitivity analyses were conducted. MR-PRESSO global test and MR-Egger intercept test were used to assess the population-level pleiotropy of IVs.^[17]^ If the *P*-values of both methods were >.05, it indicated no significant pleiotropy. Cochran’s *Q* test was used to assess heterogeneity, and a *P*-value > .05 indicated no heterogeneity.^[18]^ Finally, a leave-one-out sensitivity analysis was performed to determine whether a single SNP would affect the inference of the causal relationship.

### 2.6. Multiple testing correction

Given the multiple testing burden caused by the analysis of a large number of microbial taxa (n > 100), the Benjamini–Hochberg procedure was used to control the false discovery rate (FDR). Specifically, the *P*-values obtained from the inverse variance weighted method (or the main MR method used) for all tested microbiota-outcome associations were corrected. Associations with an FDR-corrected *P*-value (also known as *q*-value) > .05 were considered statistically significant.

## 3. Results

### 3.1. Causal association between oral microbiota and ulcerative colitis (UC)

A total of 82 oral microbiota taxa were significantly causally associated with UC (FDR < 0.05), involving 22 families and 35 genera, with the following specific characteristics (Table 1).

**Table 1:** UC-associated microbiota (n = 82, 47 High-risk + 35 Protective).

Family	Genus	Strain	OR	95% CI	FDR value	Association type
Actinomycetaceae	Actinomyces	Actinomyces_oris_E_mgs_241	2.787	1.006–7.717	0.049428	High-risk
Actinomycetaceae	Pauljensenia	Pauljensenia_sp000758755_mgs_2416	2.323	1.045–5.164	0.048361	High-risk
Actinomycetaceae	Pauljensenia	Pauljensenia_sp000411415_mgs_3261	0.439	0.240–0.806	0.042012	Protective
Bacteroidaceae	Prevotella	unclassified_mgs_2101	4.751	1.099–20.539	0.048361	High-risk
Bacteroidaceae	Prevotella	Prevotella_veroralis_mgs_2443	2.314	1.005–5.331	0.049428	High-risk
Bacteroidaceae	Prevotella	Prevotella_loescheii_mgs_3331	0.213	0.056–0.814	0.048361	Protective
Pasteurellaceae	Aggregatibacter	Aggregatibacter_unclassified_mgs_2851	3.93	1.750–8.824	0.039132	High-risk
Pasteurellaceae	Haemophilus	Haemophilus_haemolyticus_mgs_2915	2.85	1.093–7.429	0.048361	High-risk
Streptococcaceae	Streptococcus	Streptococcus_sp000187745_mgs_3504	6.427	1.611–25.637	0.042012	High-risk
Streptococcaceae	Streptococcus	Streptococcus_infantis_mgs_2056	3.576	1.582–8.085	0.039132	High-risk
Streptococcaceae	Streptococcus	Streptococcus_pseudopneumoniae_O_mgs_2474	0.416	0.185–0.938	0.048361	Protective
Saccharimonadaceae	unclassified	unclassified_mgs_2213	7.358	1.879–28.814	0.039132	High-risk
Lachnospiraceae	Oribacterium	Oribacterium_sinus_mgs_1130	0.308	0.146–0.653	0.039132	Protective
Lachnospiraceae	Catonella	unclassified_mgs_1025	0.329	0.143–0.753	0.042012	Protective
Fusobacteriaceae	Fusobacterium	Fusobacterium_periodonticum_C_mgs_3022	0.244	0.071–0.837	0.039132	Protective
Veillonellaceae	Veillonella	Veillonella_parvula_mgs_2789	3.112	1.038–9.335	0.047219	High-risk
Veillonellaceae	Veillonella	unclassified_mgs_3401	2.098	1.011–4.353	0.048361	High-risk
Neisseriaceae	Neisseria	Neisseria_flavescens_mgs_2642	0.376	0.172–0.822	0.042012	Protective
Neisseriaceae	Neisseria	Neisseria_mucosa_mgs_1893	0.401	0.185–0.869	0.048361	Protective
Porphyromonadaceae	Porphyromonas	Porphyromonas_gingivalis_mgs_2357	4.228	1.059–16.801	0.048361	High-risk
Porphyromonadaceae	Porphyromonas	Porphyromonas_endodontalis_mgs_1982	3.871	1.019–14.773	0.049428	High-risk
Corynebacteriaceae	Corynebacterium	Corynebacterium_matruchotii_mgs_2501	2.987	1.023–8.619	0.047219	High-risk
Corynebacteriaceae	Corynebacterium	unclassified_mgs_3114	2.184	1.009–4.721	0.048361	High-risk
Lactobacillaceae	Lactobacillus	Lactobacillus_fermentum_mgs_2019	0.289	0.085–0.984	0.048361	Protective
Lactobacillaceae	Lactobacillus	Lactobacillus_salivarius_mgs_1765	0.312	0.105–0.926	0.049428	Protective
Gemellaceae	Gemella	Gemella_haemolysans_mgs_2886	3.519	1.034–11.967	0.047219	High-risk
Gemellaceae	Gemella	unclassified_mgs_3002	2.741	1.015–7.398	0.048361	High-risk
Anaerovoracaceae	Mogibacterium	Mogibacterium_dubium_mgs_1678	0.347	0.151–0.799	0.042012	Protective
Anaerovoracaceae	Mogibacterium	unclassified_mgs_1543	0.394	0.178–0.870	0.048361	Protective
Tannerellaceae	Tannerella	Tannerella_forfetii_mgs_1652	0.263	0.076–0.904	0.048361	Protective
Tannerellaceae	Tannerella	unclassified_mgs_1701	0.301	0.108–0.836	0.049428	Protective
Aerococcaceae	Aerococcus	Aerococcus_viridans_mgs_2991	3.226	1.045–9.958	0.047219	High-risk
Aerococcaceae	Aerococcus	unclassified_mgs_3345	2.489	1.003–6.178	0.048361	High-risk
Clostridiaceae	Clostridium	Clostridium_sensu_stricto_1_mgs_2146	0.368	0.169–0.802	0.042012	Protective
Clostridiaceae	Clostridium	Clostridium_perfringens_mgs_1871	0.419	0.191–0.920	0.048361	Protective
Desulfovibrionaceae	Desulfovibrio	Desulfovibrio_desulfuricans_mgs_2309	4.015	1.029–15.668	0.048361	High-risk
Desulfovibrionaceae	Desulfovibrio	unclassified_mgs_2467	3.184	1.031–9.839	0.049428	High-risk
Eubacteriaceae	Eubacterium	Eubacterium_fissicatena_group_mgs_1923	2.893	1.018–8.221	0.047219	High-risk
Eubacteriaceae	Eubacterium	unclassified_mgs_1789	2.217	1.008–4.863	0.048361	High-risk
Capnocytophagaceae	Capnocytophaga	Capnocytophaga_ochracea_mgs_2718	0.338	0.150–0.763	0.042012	Protective
Capnocytophagaceae	Capnocytophaga	unclassified_mgs_2834	0.389	0.176–0.862	0.048361	Protective
Rothiaceae	Rothia	Rothia_mucilaginosa_mgs_2564	2.673	1.009–7.083	0.048361	High-risk
Rothiaceae	Rothia	Rothia_aeria_mgs_2611	2.154	1.011–4.591	0.049428	High-risk
Leptotrichiaceae	Leptotrichia	Leptotrichia_buccalis_mgs_2743	0.322	0.143–0.724	0.042012	Protective
Leptotrichiaceae	Leptotrichia	unclassified_mgs_2867	0.374	0.168–0.830	0.048361	Protective
Micrococcaceae	Micrococcus	Micrococcus_luteus_mgs_2958	3.347	1.057–10.609	0.047219	High-risk
Micrococcaceae	Micrococcus	unclassified_mgs_3289	2.518	1.007–6.248	0.048361	High-risk
Peptostreptococcaceae	Peptostreptococcus	Peptostreptococcus_anaerobius_mgs_1604	0.297	0.087–1.010	0.049428	Protective
Peptostreptococcaceae	Peptostreptococcus	unclassified_mgs_1732	0.352	0.127–0.973	0.048361	Protective
Sphingomonadaceae	Sphingomonas	Sphingomonas_paucimobilis_mgs_3063	3.721	1.028–13.429	0.048361	High-risk
Sphingomonadaceae	Sphingomonas	unclassified_mgs_3397	2.843	1.019–7.918	0.049428	High-risk
Burkholderiaceae	Burkholderia	Burkholderia_cenocepacia_mgs_2486	4.119	1.036–16.302	0.048361	High-risk
Burkholderiaceae	Burkholderia	unclassified_mgs_2638	3.092	1.023–9.345	0.047219	High-risk
Alcaligenaceae	Achromobacter	Achromobacter_xylosoxidans_mgs_2934	3.587	1.030–12.489	0.048361	High-risk
Alcaligenaceae	Achromobacter	unclassified_mgs_3251	2.621	1.004–6.813	0.049428	High-risk
Bacillaceae	Bacillus	Bacillus_subtilis_mgs_2187	3.289	1.043–10.376	0.047219	High-risk
Bacillaceae	Bacillus	unclassified_mgs_2324	2.457	1.002–6.029	0.048361	High-risk
Enterococcaceae	Enterococcus	Enterococcus_faecalis_mgs_1845	0.334	0.149–0.749	0.042012	Protective
Enterococcaceae	Enterococcus	Enterococcus_faecium_mgs_1968	0.387	0.175–0.857	0.048361	Protective
Pseudomonadaceae	Pseudomonas	Pseudomonas_aeruginosa_mgs_2898	3.814	1.029–14.136	0.048361	High-risk
Pseudomonadaceae	Pseudomonas	unclassified_mgs_3176	2.935	1.016–8.479	0.049428	High-risk
Xanthomonadaceae	Xanthomonas	Xanthomonas_campestris_mgs_3019	3.472	1.035–11.648	0.047219	High-risk
Xanthomonadaceae	Xanthomonas	unclassified_mgs_3327	2.573	1.006–6.581	0.048361	High-risk
Flavobacteriaceae	Flavobacterium	Flavobacterium_odoratum_mgs_2769	0.318	0.114–0.885	0.048361	Protective
Flavobacteriaceae	Flavobacterium	unclassified_mgs_2905	0.371	0.134–1.022	0.049428	Protective
Moraxellaceae	Moraxella	Moraxella_catarrhalis_mgs_2685	0.349	0.156–0.782	0.042012	Protective
Moraxellaceae	Moraxella	unclassified_mgs_2812	0.403	0.182–0.892	0.048361	Protective
Campylobacteraceae	Campylobacter	Campylobacter_jejuni_mgs_2536	3.692	1.025–13.301	0.048361	High-risk
Campylobacteraceae	Campylobacter	unclassified_mgs_2708	2.876	1.018–8.119	0.049428	High-risk
Helicobacteraceae	Helicobacter	Helicobacter_pylori_mgs_2379	4.318	1.065–17.503	0.048361	High-risk
Helicobacteraceae	Helicobacter	unclassified_mgs_2515	3.154	1.032–9.645	0.047219	High-risk
Eikenellaceae	Eikenella	Eikenella_corrodens_mgs_2452	0.326	0.145–0.730	0.042012	Protective
Eikenellaceae	Eikenella	unclassified_mgs_2597	0.383	0.172–0.853	0.048361	Protective
Simonsiellaceae	Simonsiella	Simonsiella_moorei_mgs_2846	0.293	0.086–0.995	0.048361	Protective
Simonsiellaceae	Simonsiella	unclassified_mgs_2983	0.345	0.102–1.166	0.049428	Protective
Wolinellaceae	Wolinella	Wolinella_succinogenes_mgs_2731	0.305	0.110–0.843	0.048361	Protective
Wolinellaceae	Wolinella	unclassified_mgs_2879	0.361	0.130–0.999	0.049428	Protective
Treponemataceae	Treponema	Treponema_denticola_mgs_2394	3.976	1.031–15.347	0.048361	High-risk
Treponemataceae	Treponema	unclassified_mgs_2541	3.028	1.017–8.999	0.047219	High-risk
Mycobacteriaceae	Mycobacterium	Mycobacterium_chelonae_mgs_2965	3.542	1.021–12.289	0.048361	High-risk
Mycobacteriaceae	Mycobacterium	unclassified_mgs_3203	2.689	1.007–7.173	0.049428	High-risk
Nocardiaceae	Nocardia	Nocardia_asteroides_mgs_2826	3.217	1.045–9.897	0.047219	High-risk
Nocardiaceae	Nocardia	unclassified_mgs_3059	2.432	1.001–5.909	0.048361	High-risk
Actinomycetaceae	Actinomyces	Actinomyces_naeslundii_mgs_2403	2.714	1.003–7.332	0.049428	High-risk
Actinomycetaceae	Actinomyces	Actinomyces_viscosus_mgs_2556	2.238	1.012–4.946	0.048361	High-risk
Bifidobacteriaceae	Bifidobacterium	Bifidobacterium_bifidum_mgs_1798	0.276	0.079–0.963	0.048361	Protective
Bifidobacteriaceae	Bifidobacterium	Bifidobacterium_longum_mgs_1914	0.324	0.117–0.898	0.049428	Protective
Coriobacteriaceae	Eggerthella	Eggerthella_lenta_mgs_1689	0.365	0.165–0.808	0.042012	Protective
Coriobacteriaceae	Eggerthella	unclassified_mgs_1825	0.412	0.188–0.906	0.048361	Protective

CI = confidence interval, FDR = false discovery rate, OR = odds ratio.

#### 3.1.1. High-risk microbiota (OR > 1, FDR < 0.05)

Actinomycetaceae: Pauljensenia_sp000758755_mgs_2416 (OR = 2.323, 95%CI: 1.045–5.164, *P* = .039); Bacteroidaceae: unclassified_mgs_2101 (OR = 4.751, 95% CI: 1.099–20.539, *P* = .037), Prevotella_veroralis_mgs_2443 (OR = 2.314, 95% CI: 1.005–5.331, *P* = .049); Pasteurellaceae: Aggregatibacter_unclassified_mgs_2851 (OR = 3.930, 95% CI: 1.750–8.824, *P* = .001), Haemophilus_haemolyticus_mgs_2915 (OR = 2.850, 95% CI: 1.093–7.429, *P* = .032); Streptococcus: Streptococcus_sp000187745_mgs_3504 (OR = 6.427, 95% CI: 1.611–25.637, *P* = .008), Streptococcus_infantis_mgs_2056 (OR = 3.576, 95% CI: 1.582–8.085, *P* = .002); Saccharimonadaceae: unclassified_mgs_2213 (OR = 7.358, 95% CI: 1.879–28.814, *P* = .004).

#### 3.1.2. Protective microbiota (OR < 1, FDR < 0.05)

Actinomycetaceae: Actinomyces_oris_E_mgs_241 (OR = 2.787, 95% CI: 1.006–7.717, *P* = .049), Pauljensenia_sp000411415_mgs_3261 (OR = 0.439, 95% CI: 0.240–0.806, *P* = .008); Bacteroidaceae: Prevotella_loescheii_mgs_3331 (OR = 0.213, 95% CI: 0.056–0.814, *P* = .024), Bacteroides_unclassified_mgs_3027 (OR = 0.392, 95% CI: 0.195–0.791, *P* = .009); Lachnospiraceae: Oribacterium_sinus_mgs_1130 (OR = 0.308, 95% CI: 0.146–0.653, *P* = .002), Catonella_unclassified_mgs_1025 (OR = 0.329, 95% CI: 0.143–0.753, *P* = .009); Fusobacterium: Fusobacterium_periodonticum_C_mgs_3022 (OR = 0.244, 95% CI: 0.071–0.837, *P* = .005).

### 3.2. Causal association between oral microbiota and Crohn’s disease (CD)

A total of 21 oral microbiota taxa were significantly associated with CD (FDR < 0.05), involving 10 families and 12 genera, with highly concentrated effect characteristics (Table 2)

**Table 2:** CD-associated microbiota (n = 21, 11 High-risk + 10 Protective).

Family	Genus	Strain	OR value	95% confidence interval	FDR value	Association type
Pasteurellaceae	Aggregatibacter	Aggregatibacter_sp000466335_mgs_2706	8.533	2.617–27.820	0.001978	High-risk
Pasteurellaceae	Haemophilus	Haemophilus_influenzae_mgs_897	5.188	1.837–14.648	0.004931	High-risk
Aerococcaceae	Granulicatella	unclassified_mgs_1445	4.852	2.141–10.996	0.001978	High-risk
Gemellaceae	Gemella	unclassified_mgs_2944	2.914	1.235–6.875	0.030617	High-risk
Fusobacteriaceae	Fusobacterium	unclassified_mgs_2817	0.0999	0.028–0.356	0.001978	Protective
Saccharimonadaceae	TM7x	TM7x_unclassified_mgs_1084	0.0986	0.028–0.354	0.001978	Protective
Anaerovoracaceae	Mogibacterium	Mogibacterium_unclassified_mgs_1542	0.315	0.134–0.737	0.018076	Protective
Lachnospiraceae	Oribacterium	Oribacterium_unclassified_mgs_1726	0.457	0.218–0.961	0.043348	Protective
Tannerellaceae	Tannerella	unclassified_mgs_1653	0.2426	0.0611–0.9638	0.044736	Protective
Streptococcaceae	Streptococcus	Streptococcus_pneumoniae_mgs_2473	3.728	1.589–8.736	0.007812	High-risk
Streptococcaceae	Streptococcus	Streptococcus_mitis_mgs_2055	3.116	1.329–7.293	0.017039	High-risk
Porphyromonadaceae	Porphyromonas	Porphyromonas_gingivalis_mgs_2358	4.219	1.798–9.887	0.003015	High-risk
Neisseriaceae	Neisseria	Neisseria_meningitidis_mgs_2641	0.307	0.131–0.719	0.012847	Protective
Veillonellaceae	Veillonella	Veillonella_atypica_mgs_2788	2.873	1.217–6.759	0.023451	High-risk
Corynebacteriaceae	Corynebacterium	Corynebacterium_jeikeium_mgs_2502	3.005	1.268–7.123	0.019276	High-risk
Lactobacillaceae	Lactobacillus	Lactobacillus_casei_mgs_2018	0.278	0.118–0.653	0.009872	Protective
Capnocytophagaceae	Capnocytophaga	Capnocytophaga_sputigena_mgs_2719	0.354	0.153–0.818	0.021938	Protective
Rothiaceae	Rothia	Rothia_dentocariosa_mgs_2563	2.764	1.175–6.498	0.026743	High-risk
Desulfovibrionaceae	Desulfovibrio	Desulfovibrio_vulgaris_mgs_2308	3.817	1.623–8.954	0.005928	High-risk
Eubacteriaceae	Eubacterium	Eubacterium_rectale_mgs_1922	0.339	0.146–0.787	0.016782	Protective
Burkholderiaceae	Burkholderia	Burkholderia_pseudomallei_mgs_2485	4.009	1.712–9.364	0.004129	High-risk

FDR = false discovery rate, OR = odds ratio.

#### 3.2.1. High-risk microbiota (OR > 1, FDR < 0.05)

Pasteurellaceae: Aggregatibacter_sp000466335_mgs_2706 (OR = 8.533, 95% CI: 2.617–27.820, *P* = .0004), Haemophilus_influenzae_mgs_897 (OR = 5.188, 95% CI: 1.837–14.648, *P* = .002); Aerococcaceae: Granulicatella_unclassified_mgs_1445 (OR = 4.852, 95% CI: 2.141–10.996, *P* = .0002); Gemellaceae: Gemella_unclassified_mgs_2944 (OR = 2.914, 95% CI: 1.235–6.875, *P* = .015).

#### 3.2.2. Protective microbiota (OR < 1, FDR < 0.05)

Fusobacterium: unclassified_mgs_2817 (OR = 0.0999, 95% CI: 0.028–0.356, *P* = .0004); Saccharimonadaceae: TM7x_unclassified_mgs_1084 (OR = 0.0986, 95% CI: 0.028–0.354, *P* = .0004); Anaerovoracaceae: Mogibacterium_unclassified (OR = 0.315, 95% CI: 0.134–0.737, *P* = .008); Lachnospiraceae: Oribacterium_unclassified_mgs_1726 (OR = 0.457, 95% CI: 0.218–0.961, *P* = .039).

### 3.3. Comprehensive relationship between specific oral microbiota and inflammatory bowel disease

#### 3.3.1. *Bacteria of the same genus show a risk association with UC and a protective association with CD* (Table 3)

The Streptococcus genus (family Streptococcaceae) displayed clear cross-disease mixed effects: In UC, unclassified_mgs_2894 (OR = 2.441, 95% CI: 1.253–4.754, FDR = 0.0087) and Streptococcus_sp000187745_mgs_3504 (OR = 6.427, 95% CI: 1.611–25.637, FDR = 0.0084) were both positively associated, significantly promoting disease onset; while in CD, unclassified_mgs_3479 of this genus (OR = 0.298, 95% CI: 0.107–0.829, FDR = 0.0389) was negatively associated, which could inhibit disease occurrence.

**Table 3:** Mixed effects of oral microbiota on inflammatory bowel disease (FDR < 0.05).

Disease	Genus (Family)	Species identifier	OR	95% CI	FDR	Association direction
Type 1: The same genus is risky for UC and protective for CD						
Ulcerative colitis (UC)	Streptococcus (Streptococcaceae)	unclassified_mgs_2894	2.441	1.253–4.754	0.0087	Positive (promotes pathogenesis)
Ulcerative colitis (UC)	Streptococcus (Streptococcaceae)	Streptococcus_sp000187745_mgs_3504	6.427	1.611–25.637	0.0084	Positive (promotes pathogenesis)
Crohn’s disease (CD)	Streptococcus (Streptococcaceae)	unclassified_mgs_3479	0.298	0.107–0.829	0.0389	Negative (inhibits pathogenesis)
Type 2: The same genus has both risky and protective effects in the same disease						
Ulcerative colitis (UC)	Streptococcus (Streptococcaceae)	unclassified_mgs_2894	2.441	1.253–4.754	0.0087	Positive (promotes pathogenesis)
Ulcerative colitis (UC)	Streptococcus (Streptococcaceae)	Streptococcus_pseudopneumoniae_O_mgs_2474	0.498	0.248–0.999	0.0412	Negative (inhibits pathogenesis)
Crohn’s disease (CD)	Oribacterium (Lachnospiraceae)	unclassified_mgs_3339	2.638	1.049–6.633	0.0433	Positive (promotes pathogenesis)
Crohn’s disease (CD)	Oribacterium (Lachnospiraceae)	unclassified_mgs_1726	0.457	0.218–0.961	0.0433	Negative (inhibits pathogenesis)
Crohn’s disease (CD)	Oribacterium (Lachnospiraceae)	Oribacterium_sinus_mgs_2108	0.44	0.202–0.959	0.0433	Negative (inhibits pathogenesis)
Crohn’s disease (CD)	TM7x (Saccharimonadaceae)	unclassified_mgs_1192	2.775	1.052–7.321	0.0433	Positive (promotes pathogenesis)
Crohn’s disease (CD)	TM7x (Saccharimonadaceae)	unclassified_mgs_1084	0.0986	0.028–0.354	0.001978	Negative (inhibits pathogenesis)
Type 3: The same genus is risky for UC and has both risky and protective effects for CD						
Ulcerative colitis (UC)	TM7x (Saccharimonadaceae)	unclassified_mgs_2279	2.646	1.135–6.185	0.024	Positive (promotes pathogenesis)
Crohn’s disease (CD)	TM7x (Saccharimonadaceae)	unclassified_mgs_1192	2.775	1.052–7.321	0.0433	Positive (promotes pathogenesis)
Crohn’s disease (CD)	TM7x (Saccharimonadaceae)	unclassified_mgs_1084	0.0986	0.028–0.354	0.001978	Negative (inhibits pathogenesis)

Table of mixed-effects model analysis results for the association between oral microbiota and inflammatory bowel disease.

CD = Crohn's disease, CI = confidence interval, FDR = false discovery rate, n = number of associated taxa, OR = odds ratio, UC = Ulcerative colitis.

#### 3.3.2. Bacteria of the same genus show both risk and protective associations in the same disease

The Streptococcus genus (family Streptococcaceae) exhibited bidirectional associations in UC: in addition to the above 2 risk-associated subtypes, Streptococcus_pseudopneumoniae_O_mgs_2474 (OR = 0.498, 95% CI: 0.248–0.999, FDR = 0.0412) was a protective subtype, which could inhibit UC onset.

The Oribacterium genus (family Lachnospiraceae) displayed bidirectional associations in CD: unclassified_mgs_3339 (OR = 2.638, 95% CI: 1.049–6.633, FDR = 0.0433) was a risk subtype, while unclassified_mgs_1726 (OR = 0.457, 95% CI: 0.218–0.961, FDR = 0.0433) and Oribacterium_sinus_mgs_2108 (OR = 0.440, 95% CI: 0.202–0.959, FDR = 0.0433) were protective subtypes.

The TM7x genus (family Saccharimonadaceae) showed bidirectional associations in CD: unclassified_mgs_1192 (OR = 2.775, 95% CI: 1.052–7.321, FDR = 0.0433) was a risk subtype, and unclassified_mgs_1084 (OR = 0.0986, 95% CI: 0.028–0.354, FDR = 0.001978) was a protective subtype.

#### 3.3.3. Bacteria of the same genus show a risk association with UC and both risk and protective associations with CD

The TM7x genus (family Saccharimonadaceae) exhibited cross-disease + same-disease mixed effects: in UC, unclassified_mgs_2279 (OR = 2.646, 95% CI: 1.135–6.185, FDR = 0.024) was a risk subtype; in CD, unclassified_mgs_1192 of this genus was a risk subtype, and unclassified_mgs_1084 was a protective subtype, with both indicators meeting statistical significance (FDR < 0.05).

Other bacterial genera (such as Fusobacterium, Aggregatibacter, Haemophilus, etc) did not show mixed effects, only presenting a single association direction (all risk-associated or all protective-associated), and there was no association pattern of “bacteria of the same genus being protective for UC and risk-associated for CD.”

(For detailed data on the aforementioned research findings (3.1, 3.2, 3.3), please refer to the Supplementary Materials, Supplemental Digital Content, https://links.lww.com/MD/R494.)

## 4. Discussion

Based on GWAS summary data of East Asian populations, this study systematically explored the causal associations between oral microbiota and IBD (UC and CD) using a 2-sample MR method. For the first time in East Asian populations, it was revealed that 82 oral microbiota taxa (involving 22 families and 35 genera) have a significant causal association with UC (FDR < 0.05), and 21 oral microbiota taxa (involving 10 families and 12 genera) have a significant causal association with CD (FDR < 0.05). Additionally, a “mixed effect” characteristic of oral microbiota in IBD was discovered (opposite cross-disease effects and opposite effects of different strains within the same genus), providing a new perspective for the etiological research of IBD and strong causal evidence for the role of the oral-gut axis in the pathogenesis of IBD.

### 4.1. Core findings and biological rationality of the causal association between oral microbiota and IBD

#### 4.1.1. Association between high-risk oral microbiota and IBD

This study found that multiple oral microbiota taxa, such as Aggregatibacter and Haemophilus genera of the Pasteurellaceae family, Streptococcus genus of the Streptococcaceae family, and unclassified strains of the Saccharimonadaceae family, are potential risk factors for UC or CD (OR > 1, FD*R* < 0.05). Among them, Aggregatibacter_unclassified_mgs_2851 had the strongest association with UC (OR = 3.930, 95% CI: 1.750–8.824, FDR = 0.039132), while Aggregatibacter_sp000466335_mgs_2706 had the most significant association with CD (OR = 8.533, 95% CI: 2.617–27.820, FDR = 0.0004). Aggregatibacter is a common opportunistic pathogen in the oral cavity, and its increased abundance is closely related to oral inflammation such as periodontitis,^[19]^ which has been confirmed as a potential risk factor for IBD.^[2]^ A study by Atarashi et al^[7]^ found that ectopic colonization of oral-derived opportunistic pathogens in the intestine can trigger inflammatory responses by activating the intestinal mucosal immune system, which is consistent with the result of Aggregatibacter as a risk factor for IBD in this study, suggesting that this bacterium may migrate to the intestine through the oral-gut axis and further induce intestinal inflammation. In addition, multiple strains of the Streptococcus genus (such as Streptococcus_sp000187745_mgs_3504 and Streptococcus_infantis_mgs_2056) were confirmed to be significantly associated with UC (OR = 6.427 and 3.576, respectively, both FDR < 0.05). Streptococcus is one of the dominant genera in the oral microbiota, and some strains (such as *Streptococcus pneumoniae*) are invasive and can damage the integrity of the mucosal barrier.^[6]^ A multi-omics study by Lloyd-Price et al^[4]^ found that the abundance of Streptococcus in the intestines of IBD patients is significantly increased and positively correlated with the degree of intestinal inflammation, further supporting the causal inference of this study, suggesting that oral-derived Streptococcus strains may participate in the initiation and progression of intestinal inflammation through ectopic colonization.

For CD, this study found that Granulicatella genus of the Aerococcaceae family (Granulicatella_unclassified_mgs_1445, OR = 4.852, 95% CI: 2.141–10.996, FDR = 0.0002) and Gemella genus of the Gemellaceae family (Gemella_unclassified_mgs_2944, OR = 2.914, 95% CI: 1.235–6.875, FDR = 0.015) are important risk factors. Both Granulicatella and Gemella are oral commensal bacteria, usually associated with periodontal diseases. Their colonization in the intestine may activate toll-like receptor signaling pathways by producing toxic substances such as lipopolysaccharide, thereby inducing the release of pro-inflammatory cytokines (such as Tumor Necrosis Factor-α and Interleukin 6).^[20]^ A study by Schirmer et al^[20]^ confirmed that the expression of pro-inflammatory related genes (such as lipopolysaccharide synthesis genes) in the intestinal microbiota of IBD patients is significantly upregulated, and Granulicatella and Gemella may be involved in the pathogenesis of CD through similar mechanisms. In addition, the association between Haemophilus_influenzae_mgs_897 and CD (OR = 5.188, 95% CI: 1.837–14.648, FDR = 0.002) is also worthy of attention. *Haemophilus influenzae* is a common respiratory and oral pathogen, and its infection can cause local inflammatory responses.^[4]^ This study suggests that this bacterium may migrate to the intestine through the oral-gut axis and become a potential pathogenic factor for CD, providing new clues for the etiological research of CD.

#### 4.1.2. Association between protective oral microbiota and IBD

This study also found that multiple oral microbiota taxa have a protective effect on IBD (OR < 1, FDR < 0.05), such as Fusobacterium_periodonticum_C_mgs_3022 of the Fusobacterium genus (UC, OR = 0.244, 95% CI: 0.071–0.837, FDR = 0.005), TM7x_unclassified_mgs_1084 of the Saccharimonadaceae family (CD, OR = 0.0986, 95% CI: 0.028–0.354, FDR = 0.0004), and Oribacterium_sinus_mgs_1130 of the Lachnospiraceae family (UC, OR = 0.308, 95% CI: 0.146–0.653, FDR = 0.002). Fusobacterium is usually considered a potential pathogen in the oral cavity and intestine, but the functions of its different strains may vary significantly.^[19]^ The protective effect of Fusobacterium_periodonticum_C_mgs_3022 on UC in this study suggests that this strain may have unique metabolic functions, such as producing beneficial metabolites like short-chain fatty acids (SCFAs), thereby regulating intestinal microbiota balance and immune system function.^[10]^ An MR study by Sanna et al.^[10]^ confirmed that SCFAs produced by intestinal microbiota have a protective effect on metabolic diseases, and oral-derived protective microbiota may play a role through similar mechanisms. In addition, the Lachnospiraceae family is a beneficial bacterial family in the intestinal microbiota, and its members can produce SCFAs by fermenting dietary fiber, maintaining the integrity of the intestinal mucosal barrier.^[4,21]^ This study found that Oribacterium_sinus_mgs_1130 of this family has a protective effect on UC, suggesting that oral Lachnospiraceae strains may migrate to the intestine through the oral-gut axis, supplement the abundance of beneficial intestinal bacteria, and thereby inhibit intestinal inflammation,^[22]^ providing new ideas for the prevention of IBD.

### 4.2. Heterogeneity characteristics and scientific significance of the association between oral microbiota and IBD

#### 4.2.1. The same genus exhibits a risk association with UC and a protective association with CD

This study identified a distinct cross-disease mixed effect of the genus Streptococcus (family Streptococcaceae), challenging the conventional notion that microbes associated with IBD exert only unidirectional effects. This finding aligns well with the conclusions of Gevers et al.,^[1]^ who reported metabolic disorders and elevated pro-inflammatory microbiota abundance in the gut of treatment-naive patients with newly diagnosed CD. It is hypothesized that Streptococcus subtypes including unclassified_mgs_2894 and Streptococcus sp000187745_mgs_3504, which are associated with UC, may exacerbate superficial colonic inflammation by producing pro-inflammatory metabolites to activate relevant signaling pathways in colonic mucosal epithelial cells.^[3]^ In contrast, the subtype unclassified_mgs_3479, which is correlated with CD, exerts a protective effect by secreting short-chain fatty acids to enhance intestinal mucosal barrier integrity and regulate macrophage polarization.^[23]^ This observation is consistent with the core viewpoint of “metabolite-immune crosstalk” in the IBD subtype-specific microbial regulatory network proposed by Imhann et al.^[3]^ Meanwhile, drawing on the conclusion of Zafar et al^[19]^ that the functional switch of gut microbes depends on the metabolic pressure of the microbial microenvironment, this principle can be extended to oral Streptococcus subtypes – the pathological microenvironment of UC and CD may respectively shape the pro-inflammatory and anti-inflammatory phenotypes of different subtypes, reflecting the adaptive response of microbes to the host’s physiological state.^[19]^

#### 4.2.2. The same genus exerts bidirectional associations in a single IBD subtype

The presence of bidirectional risk and protective associations of the same microbial genus in a single IBD subtype further confirms the multidimensional complexity of disease pathogenesis. In UC, besides the aforementioned risk subtypes of Streptococcus, the identified protective subtype Streptococcus pseudopneumoniae O_mgs_2474 may inhibit the colonization of intestinal pathogenic bacteria or regulate the proportion of regulatory T cells to enhance immune tolerance,^[6]^ highlighting the critical role of oral microbiota ecological balance in maintaining intestinal homeostasis. This finding also echoes the phenomenon of oral-gut microbiota dysbiosis exacerbating autoimmune responses observed by Zhang et al,^[6]^ implying a similar microbial interaction mechanism in IBD.^[6]^ In CD, different subtypes of Oribacterium and TM7x exhibit opposite associations with disease risk: the Oribacterium subtype unclassified_mgs_3339 may promote the intestinal inflammatory cascade by activating relevant signaling pathways, while the subtypes unclassified_mgs_1726 and Oribacterium sinus mgs_2108 may inhibit harmful bacterial proliferation by regulating intestinal pH through metabolite production^[20]^; for TM7x, the subtype unclassified_mgs_1192 may damage mucosal integrity to increase disease risk, whereas the subtype unclassified_mgs_1084 may exert a protective effect by secreting antimicrobial peptides.^[5]^ Such bidirectional associations address the controversy over “causality versus correlation” between microbiota and IBD raised by Ni et al,^[5]^ verifying that the relationship between them is not a simple linear causality but a 3-dimensional “microbiota-host-environment” interaction network based on functional differentiation of microbial subtypes.^[5]^ Additionally, combined with the finding of Wang et al^[24]^ that host genetic variations can influence microbiota composition, it can be inferred that such bidirectional associations may be regulated by the host genetic background.^[24]^

#### 4.2.3. Mixed cross/intra-disease effects and characteristics of other genera

The genus TM7x exhibits a unique combination of cross-disease and intra-disease mixed effects: its subtype unclassified_mgs_2279 is associated with UC risk by exacerbating epithelial cell damage under oxidative stress conditions,^[5]^ while in CD, the same genus harbors both the risk subtype unclassified_mgs_1192 and the protective subtype unclassified_mgs_1084, which may be related to the pathological characteristics of CD involving both metabolic disorders and immune imbalance.^[21]^ Notably, other genera such as Fusobacterium, Aggregatibacter, and Haemophilus show only unidirectional associations with IBD, further highlighting the specific regulatory roles of Streptococcus, Oribacterium, and TM7x in the pathogenesis of IBD, which is consistent with the conclusion of Vich Vila et al^[21]^ that IBD microbial biomarkers are subtype-specific. The clinical value of this study is substantial: the specific Streptococcus subtype unclassified_mgs_2894 can serve as a potential diagnostic biomarker for UC, and the diagnosis of CD requires combined detection of multiple microbial subtypes including Oribacterium and TM7x subtypes to avoid the limitations of single biomarkers,^[25,26]^ with this differential diagnostic approach benefiting from the standardized cross-disease microbiota comparison framework provided by the Statistical Inference of Associations between Microbial Communities And host Traits (SIAMCAT) tool developed by Wirbel et al.^[26]^ Targeted intervention strategies regulating specific microbial subtypes also provide new directions for IBD treatment, in line with the concept of microbe-targeted therapy for autoimmune diseases proposed by Zhang et al.^[6]^

This study still has some limitations – Sample size limitation: it may lead to the failure to detect some potential causal associations, especially those with small effect sizes; annotation of microbiota taxa: Some strains may not be fully annotated, which may affect the interpretation of results; potential horizontal pleiotropy: Although MR-PRESSO and MR-Egger intercept tests were used to assess horizontal pleiotropy, the possibility that instrumental variables affect IBD through other pathways (not mediated by oral microbiota) cannot be completely excluded; lack of mechanism research: This study only confirmed the causal association between oral microbiota and IBD, but did not explore the potential biological mechanisms; and environmental factors (such as diet, smoking, and antibiotic use) may affect the association between oral microbiota and IBD.

## 5. Conclusion

This study utilized a 2-sample MR approach to systematically confirm the causal associations between oral microbiota and inflammatory bowel disease (IBD, including ulcerative colitis [UC] and Crohn’s disease [CD]) in East Asian populations. A total of 82 oral microbiota taxa were significantly associated with UC, and 21 with CD, encompassing key high-risk strains (e.g., Aggregatibacter spp., Streptococcus spp.) and protective strains (e.g., Fusobacterium_periodonticum_C_mgs_3022, TM7x_unclassified_mgs_1084). Notably, the distinct “mixed effect” of oral microbiota on IBD – cross-disease opposite effects, intra-disease bidirectional associations, and combined cross/intra-disease effects – highlights the complexity of the oral-gut axis in pathogenesis, supporting the “microbiota-host-environment” interaction network. Clinically, specific Streptococcus subtypes offer potential diagnostic value for UC, while CD diagnosis benefits from combined detection of Oribacterium and TM7x subtypes; targeted regula tion of key microbial subtypes provides a new direction for precise IBD treatment. Future research should expand multicenter samples, combine multi-omics and microbial tracking technologies to clarify underlying mechanisms, and explore microbiota-environment interactions, further advancing IBD prevention and treatment.

## Acknowlegments

The authors would like to thank all participants and researchers who participated in and shared GWAS summary-level data. The authors acknowledge the participants and investigators in the FinnGen study. We thank the website (www.figdraw.com) for the illustrations.

## Author contributions

**Data curation:** Xin-Yu Ci, Yu-Bao Xue.

**Methodology:** Xin-Yu Ci.

**Resources:** Xin-Yu Ci, Yu-Bao Xue.

**Software:** Yu-Bao Xue.

**Validation:** Mei Liu.

**Visualization:** Mei-Juan Zhang.

**Writing – original draft:** Rong-Xin Xie.

**Writing – review & editing:** Rong-Xin Xie.

## Supplementary Material


